# Long COVID in Children: A Multidisciplinary Review

**DOI:** 10.3390/diagnostics13121990

**Published:** 2023-06-07

**Authors:** Francesco Sansone, Giulia Michela Pellegrino, Antonio Caronni, Federica Bonazza, Elena Vegni, Alberto Lué, Tommaso Bocci, Carlotta Pipolo, Giuliano Giusti, Paola Di Filippo, Sabrina Di Pillo, Francesco Chiarelli, Giuseppe Francesco Sferrazza Papa, Marina Attanasi

**Affiliations:** 1 Unit of Paediatrics, Rovigo Hospital, 45030 Rovigo, Italy; francesco.sansone1991@gmail.com; 2 Department of Neurorehabilitation Sciences, Casa di Cura Igea, 20144 Milan, Italy; giuliampellegrino@gmail.com; 3 Department of Neurorehabilitation Sciences, IRCCS Istituto Auxologico Italiano, Ospedale San Luca, 20122 Milan, Italy; antonio.caronni@gmail.com; 4 Department of Biomedical Sciences for Health, Università degli Studi di Milano, 20133 Milan, Italy; 5 Department of Health Sciences, Clinical Psychology, University of Milan, Via di Rudinì 8, 20142 Milan, Italy; federica.bonazza@unimi.it (F.B.); elena.vegni@unimi.it (E.V.); 6 Unit of Clinical Psychology, San Paolo Hospital, ASST Santi Paolo e Carlo, Via di Rudinì 8, 20142 Milan, Italy; 7 Service of Digestive Diseases, University Clinic Hospital Lozano Blesa, IIS Aragón, 50009 Zaragoza, Spain; alberto.lue@hotmail.com; 8 Department of Health Sciences, University of Milan, 20146 Milan, Italy; tommaso.bocci@unimi.it; 9 Clinical Neurology Unit, Department of Health Sciences, “Azienda Socio-Sanitaria Territoriale Santi Paolo e Carlo”, University of Milan, 20146 Milan, Italy; 10 Department of Health Sciences, Otorhinolaryngology Department, ASST Santi Paolo e Carlo, University of Milan, 20142 Milan, Italy; carlotta.pipolo@unimi.it; 11 Paediatric Cardiology Unit, Niguarda Hospital, 20162 Milan, Italy; giuliano.giusti@ospedaleniguarda.it; 12 Paediatric Allergy and Pulmonology Unit, Department of Paediatrics, University of Chieti-Pescara, 66100 Chieti, Italy; difilippopaola@libero.it (P.D.F.);; 13 School of Medicine, University of Enna “Kore”, 94100 Enna, Italy

**Keywords:** Long COVID, children, lung function, otorhinolaryngology, gastrointestinal, psychological well-being, autonomic dysfunction, neuroCOVID

## Abstract

Long COVID syndrome has emerged as a long-lasting consequence of acute SARS-CoV-2 infection in adults. In addition, children may be affected by Long COVID, with potential clinical issues in different fields, including problems in school performance and daily activities. Yet, the pathophysiologic bases of Long COVID in children are largely unknown, and it is difficult to predict who will develop the syndrome. In this multidisciplinary clinical review, we summarise the latest scientific data regarding Long COVID and its impact on children. Special attention is given to diagnostic tests, in order to help the physicians to find potential disease markers and quantify impairment. Specifically, we assess the respiratory, upper airways, cardiac, neurologic and motor and psychological aspects. Finally, we also propose a multidisciplinary clinical approach.

## 1. Introduction

Since the beginning of the Severe Acute Respiratory Syndrome Coronavirus 2 (SARS-CoV-2) [1] pandemic, approximately 646,700,000 confirmed cases have been reported [2]. The paediatric population has been estimated to represent 8.5% of the total cases [3], reaching higher percentages in some countries such as the United States of America with 18.2% of cumulative cases [4]. The adoption of various strategies for Coronavirus Infection Disease 2019 (COVID-19) containment has helped to reduce the viral shedding and the pressure on the healthcare systems [5]. Vaccines against SARS-CoV-2 and its most important variants have significantly reduced mortality, rate of hospital admissions and disease severity [5]. Despite these advancements, many patients complain of long-lasting symptoms after recovery from COVID-19, typically involving different organs, with a waxing and waning presentation. This clinical manifestation following SARS-CoV-2 infection with no other medical explanation has been called Long COVID syndrome or Post-Acute Sequelae of COVID-19 syndrome (PASC) [6]. Recent scientific reports have raised attention to the burden of Long COVID syndrome in the paediatric population [7,8,9]. Although the clinical characteristics and the course of this condition seem to be similar to those affecting adults, there are limited data on the pathophysiologic bases [10].

We herein analyze current evidence on Long COVID syndrome in children, focusing on a clinical multidisciplinary assessment aimed at providing practical insights.

## 2. Materials and Methods

We searched for articles on PubMed and Google Scholar using the following search terms and logic for the introduction and general paragraphs on Long COVID: “COVID-19” OR “COVID” OR “SARS-CoV-2” OR “coronavirus” OR “long-COVID” OR “post COVID” AND “PASC” OR “post-acute” OR “persistent” OR “convalescent” OR “convalescence” OR “sequelae” AND “pediatric” OR “young” OR “infant” OR “children” OR “adolescents”. Additional search terms were adopted for each specialisation: for the respiratory paragraph “lung function” OR “spirometry” OR “DLCO” OR “lung ultrasound” OR “lung imaging”; for the gastrointestinal paragraph “gastrointestinal” OR “liver”; for the otorhinolaryngologic paragraph “ENT” OR “Otorhinolaryngology”; for the psychologic paragraph “psychological well-being”; for the cardiologic paragraph “Autonomic dysfunction”; for the neurologic paragraph “neuroCOVID” OR “child neurology”. Further studies were obtained through the references of some papers. Articles were selected according to their title and abstract, using eligibility criteria. The inclusion criteria were being in the English language; pediatric study population (age range 0–18 years); type of study: narrative and systematic reviews, longitudinal retrospective and prospective studies and randomised control trials, including adult studies. Additionally, case reports, expert opinions and manuscripts published in a language other than English were excluded. The final reference list was developed on the basis of originality and relevance to the broader scope of this review.

## 3. Pathophysiology

**Figure 1:**
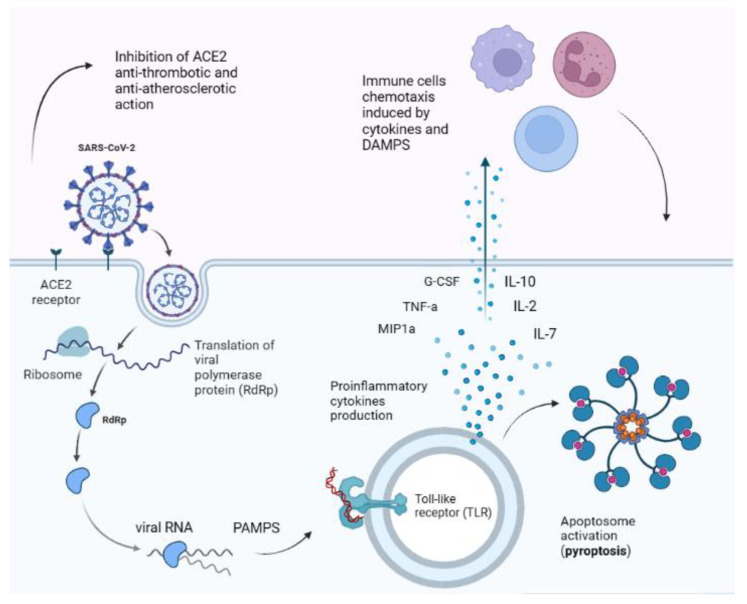
**SARS-CoV-2 main pathogenic pathways**. The virus binds to ACE2 receptor, dampening its anti-thrombotic and anti-atherosclerotic action and inducing vascular damage. Once inside the cell it replicates and viral RNA is recognised by PAMPs (pathogen-associated molecular patterns) receptor, such as Toll-like receptors (TLRs) [12,16]. Activated TLRs induce pro-inflammatory cytokines release, triggering a cascade of events that leads to cell apoptosis (pyroptosis). Cellular debris and cytokines in the bloodstream induce the activation of the innate and immune system, sustaining the inflammatory process [12]. Importantly, SARS-CoV-2 can bind to cell proteins different from ACE2 receptor, thus explaining why cells not expressing ACE2 receptor can also be infected by the virus [17,18,19,20]. Abbreviations: ACE2 = angiotensin-converting enzyme 2; PAMPs = pathogen-associated molecular patterns; DAMPS = damage-associated molecular patterns; TLR = toll-like receptor; IL = interleukin; TNF-a = tumour necrosis factor α; MIP1a = macrophage inflammatory protein 1 α; G-CSF = granulocyte-colony-stimulating factor.

There are several mechanisms that determine SARS-CoV-2 pathogenicity (Figure 1). Once inside the host cell, the viral RNA genome triggers an immune response via pathogen-associated molecular patterns (PAMPs), inducing the release of proinflammatory cytokines and an inflammatory programmed death sequence called pyroptosis [11]. The dead cells release damage-associated molecular patterns (DAMPs) into the vascular stream, recruiting migrating immune system cells, such as macrophages, monocytes and T cells [12]. In predisposed individuals, these events could lead to a “cytokine storm”, which is an uncontrolled release of pro-inflammatory cytokines, such as interleukine-2 (IL-2), IL-7, IL-10, granulocyte-colony-stimulating factor (G-CSF), tumour necrosis factor-α (TNF-α) and macrophage inflammatory protein 1α (MIP1α) [12].

It has been hypothesised that sustained activation of mast cells could be at the basis of prolonged inflammatory status in Long COVID patients, as many symptoms overlap with that of mast cell activation syndrome (MCAS), but more studies are needed to confirm this pathogenic pathway [13].

The unbalance between pro-coagulant and anti-coagulant factors during SARS-CoV-2 infection is responsible for hypoxic tissue damage in many organs. In fact, SARS-CoV-2 dampens angiotensin-converting enzyme 2 (ACE2) protective action on endothelial cells and its anti-atherosclerotic effects and impairs the fibrinolysis of amyloid fibrinogen microclots through circulating spike proteins [14,15].

T- and B-cells function is fundamental to fight SARS-CoV-2 infection. Severe patients showed low levels of interferon-γ (IFN-γ) and TNF-α and high levels of perforin and granzyme B, markers of T-cell exhaustion [21]. Antibodies production by B cells can slow down the infection by preventing S protein—ACE2 interaction or by binding to viral capsid [12]. Surprisingly, the antibody–virus complex can also facilitate the entry and replication of the virus inside phagocytic cells, a non-well understood biological mechanism called antibody-dependent enhancement (ADE) [22,23].

These molecular strategies adopted by the virus during acute infection seem to be partially responsible for the subsequent development of Long COVID syndrome. In fact, Di Sante et al. [24] found higher levels of IL-6 and IL-1 in children with PASC compared with those who recovered completely after acute infection. This aspect would demonstrate the persistence of inflammation in Long COVID children. In addition, the authors showed higher levels of plasmablasts, IgD-CD27+ memory and switched to IgM-IgD-B cells, all signs of B-cells activation [24].

Another factor which could account for symptoms persistence after acute infection is tissue and organ damage. In particular, damage to the olfactory epithelium and ciliary cells in the upper airways is responsible for persistent olfactory dysfunction, that is, anosmia and parosmia. These symptoms may persist for weeks until the olfactory mucosa is fully regenerated [15].

In a small percentage of adult patients, a pro-fibrotic mechanism is found at pulmonary level, mediated by pro-inflammatory cytokines, like IL-6 that activates fibroblasts. Nowadays, no evidence shows a similar mechanism in children [25].

SARS-CoV-2 may also spread to the central nervous system (CNS) by haematogenic or neuronal retrograde routes, causing neuroinflammation and subsequent neurological manifestations [26]. Similarly, the diffusion of the virus to the gastrointestinal tract may cause enterocytes desquamation, oedema and small bowel dilation [26]. The resulting greater permeability of the GI tract facilitates the translocation of viral proteins, such as the staphylococcal toxin-like “super antigen” segment of the spike protein, a powerful pro-inflammatory protein which is thought to be related to multisystem inflammatory syndrome in children (MIS-C) [15]. In addition, the microbiome composition is greatly affected by viral infection, with peculiar alterations which are thought to predispose inflammation in different organs (more details can be found in the following paragraph dedicated to gastrointestinal Long COVID symptoms) [15].

## 4. Long COVID in Children: Background and Definition

A first case series of five children complaining of variable long-lasting non-specific symptoms after COVID-19 in children was described in November 2020 [7]. All the children were pauci-symptomatic during the acute infection and exhibited persistent symptoms lasting 6–8 months, and one child had comorbidities and a history of peri-myocarditis after COVID-19 diagnosis [7]. After this publication, more attention was given to PASC in children. In an Italian study conducted on 129 children, the authors reported non-specific symptoms, such as fatigue, palpitations, muscle and joint pain, headache, insomnia and respiratory problems, in approximately 50% of patients 60 days or more after the infection [8]. An Australian study showed an 8% prevalence of PASC in children with asymptomatic COVID-19 infection [9]. The post-acute symptoms were mostly mild and resolved spontaneously after 8 months of follow-up [9].

PASC symptomatology is heterogeneous in both clinical presentation and the timing of exacerbation and resolution. The following definition was proposed through a Delphi-consensus process: “Post-COVID-19 condition occurs in young people with a history of confirmed SARS-CoV-2 infection, with one or more persisting physical symptoms for a minimum duration of 12 weeks after initial testing that cannot be explained by an alternative diagnosis. The symptoms have an impact on everyday functioning, may continue or develop after COVID-19 infection, and may fluctuate or relapse over time” [27].

The meta-analysis by Lopez-Leon et al. [28] offers a detailed and actual portrait of paediatric Long COVID. This work, which evaluated 21 cohort studies with a total population of 80,070, determined the persistence of Long COVID symptoms in 25.2% of children affected by acute SARS-CoV-2 infection [28]. The most common PASC-associated symptoms in children overlapped with those in adults: mood symptoms (e.g., sadness, tension, anger, depression and anxiety) (16.5%), fatigue (9.7%), sleep disorders (8.4%), headache (7.8%), respiratory symptoms (7.6%), sputum production or nasal congestion (7.5%), cognitive symptoms (e.g., less concentration, learning difficulties, confusion and memory loss) (6.3%), loss of appetite (6.1%), exercise intolerance (5.7%) and altered smell (5.6%) [28]. Many studies included in this work pointed out risk factors for Long COVID development, such as older age, female sex, severe acute-COVID-19, obesity, allergic disease and long-term health conditions [29,30,31,32]. Symptoms appeared to resolve in 54–75% of children within 1–5 months [33], with 4.4% of the cases reporting persistency at >4 weeks after COVID-19 onset and 1.8% at 8 or more weeks [32].

## 5. Long COVID Assessment in Children

### 5.1. Respiratory Assessment

In children and adolescents with Long COVID, the most frequently reported respiratory symptoms are persistent cough, fatigue and exertional dyspnoea. 

Long-term respiratory sequelae after SARS-CoV-2 infection have been thoroughly documented in the adult population and are mainly represented by physiological and radiological abnormalities. Low levels of diffusion capacity for carbon monoxide (DLCO, a marker of pulmonary vascular integrity) are the most frequently observed functional defects [34], whilst the main radiological findings are represented by interstitial abnormalities, akin to what previously described in other viral pneumonias [35]. Whilst there is emerging evidence that children may be affected by persisting functional and radiological respiratory involvement, the evidence is still limited [36,37,38,39,40].

#### 5.1.1. Pulmonary Function Tests (PFTs)

In a study evaluating lung function impairment in a paediatric population (n = 29) affected by persisting respiratory symptoms, Leftin Dobkin et al. [36] reported the most frequently observed abnormality to be exercise intolerance, assessed by the six-minute walking test (6MWT). Spirometry, plethysmography and diffusing capacity for CO (DLCO) were normal in most patients, with unremarkable chest-X ray findings [36]. A Czech multicentre study conducted on 39 adolescents similarly showed a low prevalence of functional and/or structural lung anomalies at spirometry, DLCO, 6MWT, chest X-ray and D-dimer [37]. Notably, PFTs and imaging alterations fully recovered within 1 to 8 months.

Exercise capacity abnormalities are well-characterised in the adult population. Rinaldo et al. [41] studied a cohort of 91 adult COVID-19 survivors by cardiopulmonary exercise testing, with the most frequently observed abnormality consisting in early anaerobic threshold likely induced by muscle deconditioning. The patients displayed otherwise normal PFTs and chest imaging. 

In a cohort of COVID-19 hospitalised children, Esmaeilzadeh et al. [42] found the persistence of asthma-like symptoms in 41% of the cases (n = 27). The finding is unsurprising, given the long-known link between viral infections and asthma inception and exacerbation [43]. A similar finding of obstructive respiratory disorders occurred in 27 of 60 children who underwent spirometry for persisting dyspnea after COVID-19 illness [37]. More than half of these (19) exhibited a positive bronchodilator response, thus further strengthening the suspicion of asthma.

#### 5.1.2. Thoracic Imaging

Pulmonary sequelae of COVID-19 in children have been investigated by chest X-ray and lung ultrasound (LUS). Whilst X-ray findings are mainly unremarkable, studies performed with the use of LUS identified pleural irregularities, vertical artifacts or areas of white lung and subpleural consolidations [44,45,46].

An Italian observational study conducted on a cohort of 607 children with a previous SARS-CoV-2 infection described LUS abnormal findings in a few patients, mainly pleural line irregularities (27%), B-lines (17%) and small subpleural consolidations (9%) [39]. Of note, the frequency of artifacts decreased with increasing time since infection. Patients symptomatic during the acute infection presented multiple B-lines, a non-specific pattern [47], more frequently in the symptomatic group (19% vs. 10%). Although in adults, several studies investigated the morphologic changes in lung parenchyma by using lung CT; in children, this clinical condition is limited by the ionisation radiation of the technique. Similarly, Ng et al. [48] found CT abnormalities (mostly ground-glass opacification) in 6 out of 25 asymptomatic young patients with laboratory-confirmed COVID-19 with lung involvement also in asymptomatic patients. Denina et al. [49] performed a follow-up on 25 paediatric patients 4 months after the acute infection and reported that LUS artifacts regressed over time. Recent studies used magnetic resonance imaging (MRI) to overcome the limitations of ionisation radiations [50,51,52,53,54,55,56,57,58,59,60]. Heiss et al. [40] investigated morphologic and functional changes in lung parenchyma by using low-field MRI in children with Long COVID compared with healthy controls. One subject showed linear atelectasis, whereas a large subgroup of post-acute COVID-19 participants had a significant percentage of functional defects, including ventilation, perfusion and combined defects.

In conclusion, children with Long COVID may experiment respiratory symptoms, with a tendency towards complete recovery within six months. Lung function testing is characterised by occasional, and usually mild, abnormalities and may also permit to identify ongoing coexisting asthma and other obstructive pulmonary diseases. LUS appears to be a safe, sensitive imaging technique to identify interstitial lung involvement in the paediatric population [61]. Therapeutic approaches specific to Long COVID in children other than symptomatic relief are lacking.

### 5.2. Upper Airway Assessment

Literature specifically dealing with the otorhinolaryngologic sequelae of COVID-19 in children is indeed very scant. However, the pathological long-term alterations described in other organs such as fibrotic changes due to microvascular and endothelial damage or chronic neuroinflammation are sure to be found in the Head and Neck region and may explain the vast heterogeneity of described Ear, Nose and Throat (ENT) symptoms [26]. Unsurprisingly, Miller et al. [30] in a cohort study on 5032 children showed that Long COVID could indeed affect all systems with ENT symptoms at third place after general and respiratory symptoms.

#### 5.2.1. Olfactory Disfunction 

Even though olfactory/smell impairment in the acute and early phases of COVID-19 is described anywhere between 44% and 86% of paediatric patients, the same numerosity cannot be found in those studies dealing with Long COVID [62,63]. Indeed, in the study by Roge et al. [64] in which 236 paediatric COVID-19 patients and a 142-strong comparison group were enrolled, only 12.3% of children presented with anosmia/dysgeusia, while the meta-analysis by Lopez-Leon et al. [28] attests the presence of this particular symptom (e.g., hyposmia, anosmia, hypersomnia, parosmia and phantom smell) in 5.6%, pooling 10 studies with over 2000 patients. Furthermore, the prevalence of smell disturbances is stated to be less persevering and of less duration than neuropsychological symptoms, such as headache and sleep problems [31].

#### 5.2.2. Rhinological Symptoms

As with olfactory alteration, the rhinological symptoms also could be interpreted as a persistence of acute SARS-CoV-2 infection presentation, and nasal congestion and rhinorrhoea are those most frequently encountered in Long COVID. In fact, Roge et al. [64] attest the presence of nasal congestion and rhinorrhoea in 16.1% of children with persistent symptoms.

#### 5.2.3. Otological Symptoms

Even though otitis media is found as a manifestation or associated symptom of acute COVID-19 in children [65], no literature can be found regarding longstanding infection or concomitant hearing loss in the Long COVID corollary. However, otalgia and, with it, tinnitus, vertigo and earache do present in Long COVID as found in the meta-analysis of Lopez-Leon et al. [28] and are attested at around 3% prevalence. Surely, this may be explained by the fact that Long COVID symptoms pertain more frequently to functional alterations than to persistent infectious manifestations.

#### 5.2.4. Pharyngeal Symptoms

Sore throat, dysphonia and dysphagia in children are also described in the corollary of long COVID; however, their presence in the aforementioned studies and meta-analysis is stated at around 2%, under 2% and under 1%, respectively [28,64].

Causative factors in the development of ENT Long COVID symptoms are still very much not studied, and knowledge surrounding the ENT presentation and risk factors specific to the development of Long COVID is still in its infancy. Additionally, these symptoms are by no means singular to Long COVID hindering research even further. Nonetheless, persistence after paediatric COVID-19 infection of olfactory, otological and rhinological alterations should be thoroughly assessed by the otolaryngologist. Diagnostic tools include fibroscopic evaluation and specific age-appropriate smell tests, such as the U-sniff Sniffin’ sticks. Therapeutic approaches specific to Long COVID in children other than symptomatic relief are not described.

### 5.3. Gastrointestinal Assessment

Data regarding gastrointestinal manifestations of Long COVID are scarce. According to the available information, Long COVID in children and young people shows a similar gastrointestinal manifestation pattern compared to acute disease, but the prevalence is lower [28,66,67].

In a recent multicentre cohort study including 582 patients with acute disease, the prevalence of gastrointestinal symptoms was 22% [66]. In particular, the most frequent gastrointestinal manifestations in the setting of acute disease seem to be abdominal pain (11.76%), diarrhoea (9.2%) and vomiting (5.01%) [28].

On the other hand, a recent meta-analysis showed that gastrointestinal symptoms in Long COVID have a prevalence of less than 5% in children and adolescents. Abdominal pain is the most frequent manifestation, with a pooled prevalence of 2.91% of patients, followed by constipation (2.05%), chronic diarrhoea (1.68%), nausea/vomiting (1.53%) and dysphagia (0.46%) [67].

Other manifestations, such as liver involvement, are uncommon but have a relevant clinical impact. Recently, a case series described two different patterns of liver injury presented later after a recovered SARS-CoV-2 infection [68]. The authors described two main clinical scenarios: acute liver injury with acute liver failure requiring liver transplantation and cholestatic acute hepatitis resembling the post-COVID-19 cholangiopathy reported in adults [68].

Finally, post-infectious-functional gastrointestinal disorders are common in Long COVID patients [69]. These include new-onset functional dyspepsia and irritable bowel syndrome. Nevertheless, its real prevalence in children and young people is unknown.

The pathophysiologic mechanism of gastrointestinal and hepatic injury related to COVID-19 is still debated and multiple pathways could be involved [70]. Firstly, gastrointestinal damage might be due to direct infection and the cytopathic effect of gastrointestinal cells, hepatocytes and cholangiocytes [70]. Moreover, the robust constitutive expression of ACE2 on the cholangiocytes, hepatocytes and brush border of the small intestine cells could play a role in the development of gastrointestinal manifestations leading to an altered cytokines production, persistent inflammation and increased intestinal permeability [69,70].

Moreover, COVID-19-related dysbiosis and unbalance in gut flora could affect and promote neurological, respiratory and liver manifestations via the gut–lung, gut–brain and gut–liver axes [71]. It is known that gut microbiota composition is significantly altered in patients with COVID-19 [72]. In patients with Long COVID manifestations, higher levels of Ruminococcus gnavus and Bacteroides vulgatus and lower levels of Faecalibacterium prausnitzii have been found compared with non-COVID-19 patients [71]. Furthermore, dysbiosis was persistent, lasting more than a year after the infection, because the intestine acts as a long-term reservoir. It has been demonstrated that SARS-CoV-2 RNA persists in up to 12.7% of patients at 4 months after diagnosis and in 3.8% of patients at 7 months after diagnosis [73]. Moreover, antigen persistence in the gut mucosa has been observed in patients with inflammatory disease with Long COVID symptoms [74].

Gut microbiota dysbiosis could also play a role in persistence of respiratory and neurological symptoms [71]. In this regard, Mendes-Almeida V et al. [75] demonstrated that transferring gut bacteria from patients with Long COVID to healthy mice resulted in loss of cognitive functioning and impaired lung defences in the mice, which were partially treated with probiotic.

To date, clear diagnostic criteria and protocols are not available. In children and young people, a gastrointestinal symptom could be classified as a post-COVID-19 condition if it fulfils the clinical case definition of the WHO [27]. The initial management, differential diagnosis, etiologic workup and treatment of gastrointestinal manifestation should not change with respect to non-Long COVID patients.

Preliminary results of specific treatment with micronutrients and lactoferrin for chronic gastrointestinal symptoms in children with Long COVID are available [76,77]. However, there is not sufficient evidence to establish a clear recommendation.

### 5.4. Cardiologic Assessment

In children with Long COVID, involvement of the cardiovascular system and clinical cardiovascular symptoms are constantly described in multiple studies. The five most prevalent clinical cardiovascular manifestations were orthostatic intolerance (6.9%), exercise intolerance (5.7%), chest pain (4.6%) and variations in heart rate (2.29%) [28]. Less frequently also palpitations (1.27%) were associated with children with Long COVID [28].

Interestingly, most of the cardiovascular symptoms, such as orthostatic intolerance, exercise intolerance, abnormal heart rate variability, tachycardia and palpitation, are also present in dysautonomic syndromes. Dysautonomia is a dysfunction of the autonomic nervous system and it might have a central role in the cardiovascular symptoms in patients with Long COVID. Dysautonomia could be referred to as a direct result of the SARS-CoV-2 infection or immune-mediated processes (cytokines storm), but no link has been demonstrated [78,79]. Postural orthostatic tachycardia syndrome (POTS) is a clinical condition characterised by dysautonomic dysfunction. It is characterised by a sustained heart rate increment of at least 40 beats/minute (bpm) within 10 min of standing or head-up tilt in addition to chronic orthostatic symptoms for at least 3 months duration [80]. Clinical manifestations similar to POTS have been described in adult patients with Long COVID syndrome, underlying a possible common pathogenic pathway [81]. Few cases of POTS have been described among adolescents with previous SARS-CoV-2 infection, with symptoms including light-headedness, palpitations, fatigue and generalised weakness [82,83]. The exact mechanism at the basis of POTS is still debated. Paediatric studies showed abnormally elevated levels of vasodilating hormones (such as nitric oxide, hydrogen sulphide and C-natriuretic peptide) when upright, causing splanchnic and lower extremities venous pooling [80]. In PASC-affected patients, POTS might be caused by autoantibodies against G-coupled receptors, as demonstrated in adult cases [82]. The therapeutic approach ranges from oral medications such as β-blockers or vasopressors (i.e., midodrine) to intravenous periodic infusions of normal saline for unresponsive patients [80].

Little is known regarding objective findings in children with Long COVID. In the adult, Long COVID patients with suspected cardiovascular symptomatology, ECG (electrocardiograph) aberrations (34%) like non-specific ST-T changes and conduction anomalies were found [84]. Nonetheless, converging clinical, neurophysiological and anatomopathological, evidence suggests that dysautonomia plays a key role in driving neurological complications in adulthood [85,86,87]. Among these, neurophysiology has recently highlighted a specific involvement of the efferent sympathetic activity in chronic pain syndromes following SARS-CoV-2 infection [85]. These findings are also corroborated by the observation of small-fibre loss applying in vivo corneal confocal microscopy, as well as by changes described in skin biopsies [88,89]. Moreover, anomalous findings at transthoracic echocardiography (TTE) have been reported in an adult with Long COVID [84]. TEE findings were discovered in 32% of the patients and include pericardial effusion and right ventricle dysfunction [84]. In our experience, cardiovascular Long COVID manifestations are extremely represented with considerable fluctuation in their frequency and severity.

### 5.5. Common Motor Syndromes

Long COVID in children commonly affects the motor system. According to several studies, fatigue is among the most common disturbances, being reported by approximately 10% of affected children [90], and arthralgia and muscle soreness are also frequent [91]. Even if more rarely, dizziness and even balance problems can be found in the Long COVID syndrome [28].

With respect to Refs. [92,93,94,95,96], Long COVID could even restrict children and adolescents’ participation. For example, Long COVID can affect school attendance [91], and young people cannot keep up with sports as they did before [90]. Such an effect on participation could be sustained not only by the Long COVID motor syndrome; regarding neurological impairments associated with Long COVID in children, this syndrome can also present with cognitive symptoms, which include attention and learning problems [97]. In addition, mood alterations and sleep disorders have also been reported [28].

Follow-up of children with Long COVID by a multidisciplinary team comprising physicians and rehabilitation therapists has been recommended [98,99]. However, several pieces of information are still missing about this condition [28], and motor and neurological symptoms of Long COVID in children remain to be fully understood.

The exact burden of Long COVID in children is unknown, as the prevalence of symptoms in children (motor symptoms included) varies enormously in the different studies [28,91]. Moreover, the various long COVID symptoms can have different durations, which can also vary with age [100].

In diagnostic terms, it is unclear whether fatigue and joint and muscle pain have some typical features in Long COVID. Indeed, these symptoms are not specific to Long COVID but can be found in other conditions and are common in many viral diseases. Regarding this, a recent study showed that fatigue duration in Long COVID is increased compared to other viral infections [101]. 

Protocols are unavailable to diagnose and manage Long COVID in children [99]. About therapies, treatment is usually symptomatic [90]. For example, therapeutic exercise can counteract fatigue and difficulty with physical activity [98], as in other conditions in which fatigue is the main problem.

Finally, the Long COVID effects on young people with pre-existing neurological diseases remain to be ascertained. Fatigue and learning difficulties, which, as briefly reported above, are two common neurological impairments of Long COVID, are expected to adversely impact the rehabilitation of the baseline disease [102].

### 5.6. Neurologic Assessment

#### 5.6.1. Epidemiology, Clinical Phenotypes and Their Clinical Course

Since the first description in December 2020, SARS-CoV-2 has been associated with a number of neurological complications during the disease course, as well as early manifestations at onset [103,104,105]. Three years after its beginning, the pandemic continues to teach something about its neurological involvement and possible pathways [15,106]. The incidence of neurological symptoms in children is similar to that reported in adults, as well as clinical phenotypes. However, both neurological features and putative pathogenetic mechanisms have been progressively changed over time, probably depending on different viral variants as emerged during the last years. In particular, whereas several neurological syndromes have been described in 2020, cognitive disturbances seem to characterise the last pandemic outbreaks, both in adults and children, with a focus on visuospatial abilities and executive functions [28,107,108]. Similarly, although underlying pathogenetic mechanisms are numerous and heterogeneous, they seem to be shifted from a direct viral invasion towards pro-inflammatory states and a high liability to develop autoimmunity [109].

As for adults, also for children, there is no correlation between the severity of neurological complications and the clinical course of primary respiratory disease. Moreover, a degree of clinical variability in childhood is likely to depend on the ongoing development of a child’s nervous system, with differential expression of cell receptor targets during years [110]. Up to 50% of young patients experienced headaches and an altered mental status, probably due to a multisystemic inflammatory syndrome in children [111], whereas about 5% of them developed more severe neurological complications, including seizures, encephalitis, demyelinating disorders and aseptic meningitis [112,113,114,115].

#### 5.6.2. Putative Pathogenetic Mechanisms

As described above, it has been proposed that the stronger immune response, as observed in children compared to adults with neurological COVID-19, may be due at least in part to the over–activation of microglia in young patients [110]; in this connection, it is worth noting that microglial dysregulation is associated with brain aging, neurodegeneration and a range of brain disorders [116,117,118]. In this scenario, it is worth also remembering that the receptor of the angiotensin-conversion enzyme (ACE), the main gate of entrance of SARS-CoV-2 into the cells, is highly expressed not only at the neuronal and vascular endothelial surface but also by Schwann’s cells and central oligodendrocytes [119,120]. 

Conversely, in order to support direct viral damage, a prion-like mechanism of neuroinvasion had been previously described for other coronaviruses in animals [121,122]. Recent combined neurophysiological and histopathological findings showed an early involvement of the vagus nerve and respiratory nuclei in the brainstem, probably accounting for the respiratory failure itself during the acute phases of the illness [123,124,125]. Whatever the mechanism, either direct or mediated by inflammation and autoimmunity, parenchymal involvement of the brainstem is suspected to trigger some long-term complications, such as movement disorders [126,127], including functional tic-like disorders in childhood [128].

#### 5.6.3. The Strange Case of ME/CFS and What We Can Learn about Long COVID in Children

Myalgic encephalomyelitis/chronic fatigue syndrome (ME/CFS) is a clinical condition associated with a number of viral infections, including Epstein–Barr virus (EBV) and Human Parvovirus B-19 (HPV B-19), but it has assumed a high epidemiological relevance with SARS-CoV-2, affecting millions of people worldwide [129,130,131]. It defines a long-term illness characterised by at least six months of fatigue and exhaustion, together with post-exertional malaise and unrefreshing sleep [132,133]. Before the COVID-19 pandemic, the prevalence of CFS among adolescents was estimated at 0.1–1.9% in the US, with about 13% of them experiencing a prolonged recovery [134,135]. Although the phenotype is mainly neurological, this syndrome is often accompanied by systemic features and symptoms, comprising anxiety, diarrhea and skin manifestations.

Apart from infectious illnesses, ME/CFS has been also associated with exposure to environmental toxins or stressful life events [136].

Concerning specifically ME/CFS, there are three main theories about its pathophysiology:

i. a dysregulation of the 2-5A synthetase/ribonuclease L antiviral defence pathway [137];
ii. an inhibition of the hypothalamus–pituitary–adrenal (HPA) axis, thus leading to hypocortisolism [138];
iii. an altered sympathetic nervous activity, as demonstrated by orthostatic intolerance in these patients [139].

In some cases, autoantibodies against β-adrenergic or muscarinic cholinergic receptors have also been demonstrated [140]. Many of these putative pathways are in common with Long COVID, as discussed above, especially for autonomic dysfunction.

In children, specific hospital-based rehabilitation programs for ME/CFS have been reported in detail elsewhere, which seem to be effective also when following SARS-CoV-2 infection [141,142]. In particular, cognitive behaviour therapy has been demonstrated to be effective in adolescents [143], while acceptance and commitment treatment has been proposed as an alternative [141]. Interestingly, a double-blind study using intravenous immunoglobulin in adolescents showed promising findings, but it has not been replicated so far [144]. Conversely, antiviral therapy shows no evidence of clinical benefits [145].

### 5.7. Psychological Aspects

The COVID-19 pandemic has severely threatened psychological health, as well as relational and social well-being [146,147]. Despite the lower incidence of infection and mortality in children than in adults, the negative impact of the pandemic on psychological well-being has been evident [148,149]. Children over the age of two were aware of the changes caused by the spread of the virus and were afraid of their health and family members [148]. Children experienced a state of uncertainty for a prolonged period and suffered isolation due to the closure of schools and social gathering places. Several studies have shown that the psychological pressure generated by social isolation and routine derangement increased anxiety and depression symptomatology, irritability, mood instability, behavioural and emotional changes and sleep disturbances [31,148,149,150]. Special attention should be paid to children who have been infected by COVID-19. These children were more susceptible to psychological difficulties due to the risks associated with infection, isolation and the experience of hospitalisation [151]. Moreover, these complex situations have often been compounded by the loss of a loved one and the subsequent bereavement.

Furthermore, it is noteworthy the reduction in social interaction. An Italian cohort study including children aged 4–10 years showed that, during the pandemic, children had more frequent attention-seeking behaviours and an increased need for parental closeness [152]. To attenuate the sense of loneliness, children and adolescents generally spend more time on social media and the Internet, increasing the risk of compulsive usage and explicit content access.

Overall, the COVID-19 pandemic had a significant negative impact on children’s psychosocial well-being, highlighting the need to address emotional distress. As the pandemic may have a long-term impact on the persistence of emotional reactions, it is necessary to formulate targeted interventions based on significant influencing factors.

## 6. Clinical Approach: From Diagnosis to Follow-Up

Currently, there are no guidelines for Long COVID syndrome management in children. In a recent publication, Fainardi et al. [26] proposed a schematic approach to paediatric Long COVID divided into different steps. 

The first one is represented by comprehensive medical history and physical examination. Paediatricians should actively search for Long COVID symptoms, particularly among at-risk patients, such as adolescent females and children with comorbidities. Specific symptom-based questionnaires for Long COVID may help to investigate the presence of long-lasting symptoms and their impact on everyday life. Validated scales have been proposed for adult patients, such as the symptom burden questionnaire for Long COVID (SBQ-LC) and the COVID-19 Yorkshire Rehabilitation Scale (C19-YRS) [153,154]. Unfortunately, these items require to be fulfilled directly by the patients and are not suitable for children. Other psychometric scales have been adopted to measure specific aspects of Long COVID impact on the paediatric population, most of all the psychological health status. Though useful in terms of follow-up and guidance of therapeutic interventions, none of the scales currently available are validated for assessing children.

**Figure 2:**
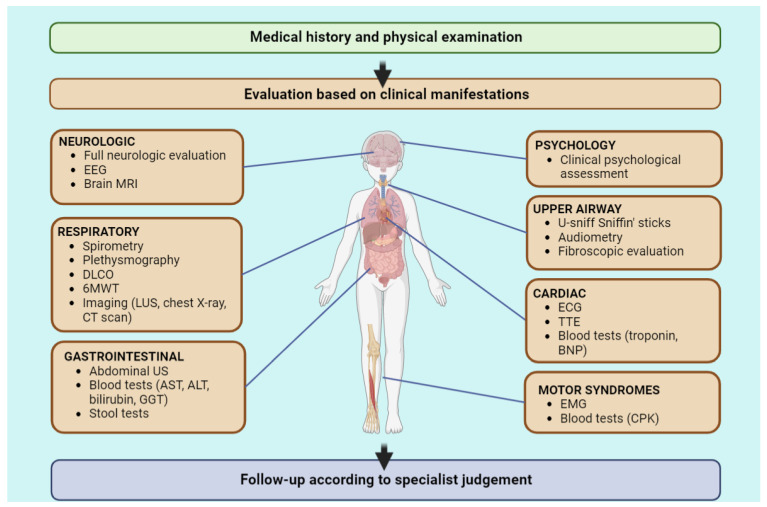
Clinical approach to paediatric Long COVID. Abbreviations: 6MWT = six-minute walking test; DLCO = diffusion lung capacity for carbon monoxide; LUS = lung ultrasound; CT = computed tomography; EMG = electromyography; CPK = creatine phosphokinase; EEG = electroencephalogram; MRI = magnetic resonance; ECG = electrocardiograph; TTE = transthoracic echocardiography; BNP = brain-derived natriuretic peptide; US = ultrasound; AST = aspartate aminotransferase; ALT = alanine aminotransferase; GGT = gamma-glutamyl transpeptidase.

Secondly, the appropriate diagnostic test should be chosen on the basis of symptoms and clinical signs (Figure 2). Non-invasive diagnostic tests, such as blood exams, PFTs, ECG, sniffing tests, audiometry and 6MWT, may be helpful to exclude the alternative diagnosis, but it should be reminded that currently there is no single diagnostic test for Long COVID. Moreover, mild abnormalities may persist also in healthy children with past SARS-CoV-2 infection and should be carefully evaluated on the basis of clinical presentation and past medical history. Non-invasive tests could be adopted for children monitoring and follow-up, as Long-COVID-related findings seem to be time-limited and generally resolve in a few months. During the third step, the monitoring phase, symptoms’ trend should be checked. Taking into account the actual knowledge, it seems reasonably advisable to leave more invasive tests (e.g., computed tomography or lung MRI) to selected cases that show persistent, atypical or worsening symptoms. 

Regarding Long COVID management, non-pharmaceutical interventions such as physical rehabilitation have been proposed to treat Long COVID symptoms in the adult population [81]. The only available randomised control trial present in the literature found that light aerobic and breath exercises improved lung function, exercise tolerance, quality of life and anxiety in a group of 72 elderly COVID-19 survivors [155]. Common analgesics, such as paracetamol and non-steroidal anti-inflammatory drugs (NSAIDs), may help to control symptoms, but there is no current effective pharmaceutical treatment for Long COVID syndrome [81]. To date, there are no available data regarding Long COVID treatment in the paediatric population. Anyway, it should be noted that this condition has a limited time course and symptoms are generally mild [26].

## 7. Conclusions

Long COVID syndrome may have a relevant impact on the daily functioning and overall quality of life of children. Symptoms are more frequently mild and generally resolve spontaneously within a few months. Currently, the pathophysiologic mechanisms are largely unknown in the paediatric population. Clinical manifestations are heterogeneous and variable over time. Available data concerning diagnostic assessment in children are mainly limited to respiratory symptoms, showing only minor pathologic findings in a subgroup of them. In the absence of permanent functional abnormalities, symptoms such as fatigue and exercise intolerance might be explained by muscle deconditioning and behavioural changes induced by lockdown and social isolation. 

Given the mild and transient disease course in most children with Long COVID, we suggest clinical follow-up along with the use of non-invasive and less dangerous diagnostic tests (i.e., LUS than chest CT for respiratory problems) as the best management options, limiting the application of more laborious tests to the few children with severe disease. Finally, paediatric Long COVID should be assessed by paediatricians and all the involved specialists in a multidisciplinary setting based on the clinical manifestations.

## Data Availability

Not applicable.
